# Global diffusion tensor imaging derived metrics differentiate glioblastoma multiforme vs. normal brains by using discriminant analysis: introduction of a novel whole-brain approach

**DOI:** 10.2478/raon-2014-0004

**Published:** 2014-04-25

**Authors:** Ernesto Roldan-Valadez, Camilo Rios, David Cortez-Conradis, Rafael Favila, Sergio Moreno-Jimenez

**Affiliations:** 1 Magnetic Resonance Unit, Medica Sur Clinic & Foundation, Mexico City, Mexico; 2 Department of Neurochemistry, National Institute of Neurology and Neurosurgery, Mexico City, Mexico; 3 GE Healthcare, Mexico city, Mexico; 4 Radioneurosurgery Unit, National Institute of Neurology and Neurosurgery, Mexico City, Mexico

**Keywords:** brain neoplasms, diffusion tensor imaging, discriminant analysis, magnetic resonance imaging, predictive value of tests

## Abstract

**Background:**

Histological behavior of glioblastoma multiforme suggests it would benefit more from a global rather than regional evaluation. A global (whole-brain) calculation of diffusion tensor imaging (DTI) derived tensor metrics offers a valid method to detect the integrity of white matter structures without missing infiltrated brain areas not seen in conventional sequences. In this study we calculated a predictive model of brain infiltration in patients with glioblastoma using global tensor metrics.

**Methods:**

Retrospective, case and control study; 11 global DTI-derived tensor metrics were calculated in 27 patients with glioblastoma multiforme and 34 controls: mean diffusivity, fractional anisotropy, pure isotropic diffusion, pure anisotropic diffusion, the total magnitude of the diffusion tensor, linear tensor, planar tensor, spherical tensor, relative anisotropy, axial diffusivity and radial diffusivity. The multivariate discriminant analysis of these variables (including age) with a diagnostic test evaluation was performed.

**Results:**

The simultaneous analysis of 732 measures from 12 continuous variables in 61 subjects revealed one discriminant model that significantly differentiated normal brains and brains with glioblastoma: Wilks’ λ = 0.324, χ^2^ (3) = 38.907, p < .001. The overall predictive accuracy was 92.7%.

**Conclusions:**

We present a phase II study introducing a novel global approach using DTI-derived biomarkers of brain impairment. The final predictive model selected only three metrics: axial diffusivity, spherical tensor and linear tensor. These metrics might be clinically applied for diagnosis, follow-up, and the study of other neurological diseases.

## Introduction

Some pathologic and magnetic resonance (MR) imaging characteristics of astrocytomas grades II to IV (highest degree known as glioblastoma multiforme, GBM) suggest these tumors would benefit from the use of a global measurement of brain impairment.^1^

The first imaging approaches to characterize high-grade glial lesions, especially GBM, were fraught with pitfalls resulting from the marked heterogeneity of both glial-infiltrated and normal brains.^2^,^3^ These tumors frequently contain multiple areas of variable histologic features, so that a sampling error in a biopsy may mean that the degree of malignancy seen by the neuropathologist may not reflect the degree of malignancy present elsewhere in the tumor, resulting in significant undergrading of some lesions.^2^ Thus, even when all radiologically visible portions of a tumor have been excised, the surgical margins may not be “clean”, and further neoplastic growth can (and usually does) occur in the adjacent brain tissue, leading from microscopic residual to gross recurrence.^4^ Therefore, none of the MR protocols for GBM in every day practice should be only morphologic.^3^,^5^,^6^ As a consequence, surgery usually only reduces the tumor; this information is relevant as recent evidence has proved gross total resection (surgical margin status) significantly correlates with progression-free, recurrence pattern and overall survival in patients with GBM.^7^,^8^

GBM is considered a whole brain disease. Radiotherapy and chemotherapy follow surgery.

Although MR perfusion and spectroscopy^9^, and sometimes diffusion tensor imaging (DTI)^10^ are routinely used methods to locate parts of the tumor-GBM with high malignancy that should be biopsied, the development of specific and sensitive biomarkers remains a critical unmet need.^11^

Our purpose in this study was to explore the diagnostic ability of a *global* (whole brain) assessment of DTI-derived tensor metrics in normal and infiltrated brains with GBM. We used the multivariate technique of linear discriminant analysis (DA), previously reported in MRI diagnosis^12^, to classify the study participants into groups, describe group differences and to assess the relative importance of DTI variables for discriminating between groups. This analysis might unveil findings and associations that cannot, in a partial-regional assessment, be recognized at surgery, neurologic, MRI and/or pathologic examination. Considering there is still scarce information in the medical literature about the global calculation of tensor metrics^13^,^14^, a predictive discriminating model may offer an innovative diagnostic approach to the surgical-neurooncology team.

## Subjects and methods

### Subjects

This was a case-control study. We included patients with suspected diagnosis and later pathological confirmation of primary GBM who had undergone preoperative brain MR examinations between January 2010 and September 2012. Exclusion criteria were corticosteroid or antibiotic treatment, lesions with areas related to calcification and/or hemorrhage and previous brain surgery. A control group included young and elderly healthy volunteers recruited from the enrolled interns and medical residents of the hospital, and elderly subjects from our Geriatrics unit. All volunteers received detailed health examinations; exclusion criteria were major neurological, psychiatric, or cardiovascular diseases. A radiologist interpreted the MR images blinded to the patient’s history and MRI examinations with other structural abnormalities were excluded. The local institutional review board approved the study (Project #2011-EXT-05).

### Brain image acquisition

MR sequences included conventional axial T2-weighted imaging, axial Fluid-Attenuated Inversion Recovery (FLAIR), axial Spoiled Gradient Echo (SPGR), DWI and axial T1-weighted imaging, using 0.1 mmol/kg of body weight of gadopentetate dimeglumine (Magnevist; Schering, Berlin, Germany). Healthy volunteers did not receive endogenous contrast. DTI was performed using a single-shot SE EPI sequence. Diffusion gradients were applied in 25 directions with b-values of 1000 s/mm^2^ and an image without diffusion weighting with b-value of 0 s/mm^2^. DTI sequences were acquired in the axial plane with 44 contiguous sections, 2.4 mm section thickness, no intersection gap; TR/TE of 17,000/80 ms, with parallel imaging to reduce off-resonance artifacts (PI factor was 2); 25 × 25 cm FOV; and 128 × 128 matrix/pixel size. MR was performed on a single occasion using a 3T unit (Signa HDxt, GE Healthcare, Waukesha, WI, USA); and a high-resolution eight-channel head coil (Invivo, Gainesville, FL, USA).

### Image postprocessing and data analysis

**FIGURE 1.**
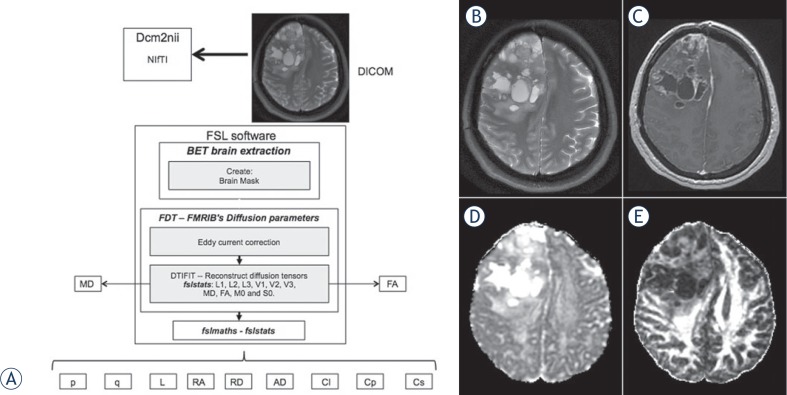
**(A),** FSL software algorithm used in the image postprocessing and data analyses. **(B–E),** Examples of acquired sequences in a patient with GBM and the tensor-metric maps generated for the data analyses: **(B),** axial T2-weighted; **(C),** post contrast axial T1-weighted; **(D),** axial diffusivity (AD) tensor map; and ** (E), ** fractional anisotropy (FA) tensor map. Notice how it might not be possible to perform an imaging diagnosis based only on a visual inspection of these maps.

We used the software dcm2nii^15^ and the FMRIB Software Library (FSL) v. 4.1.9.^16^ DTI images were extracted using the *Brain Extraction Tool (BET)* v. 2.1.^17^ Eddy currents were corrected using the *FMRIB’s Diffusion Toolbox v. 2.0;* the *Reconstruct Diffusion Tensor* (*DTIFIT)* and the *fslmaths tool* generated the eigenvector and eigenvalue maps for each tensor metric. The *fslstats tool* calculated the scalar measures (mean values) of each whole-brain calculation. The apparent diffusion coefficient (ADC) value, a simple index calculated from diffusion-weighted images^18^, was considered equivalent to the MD (mean diffusivity) metric, as it was obtained from the DTI sequence.^19^ DTI-derived tensor metrics formulas using the major (λ1), intermediate (λ2), and minor (λ3) eigenvalues allowed the calculation of the eleven most common tensor metrics for brain imaging: mean diffusivity (MD), fractional anisotropy (FA), pure isotropic diffusion (p), pure anisotropic diffusion (q), the total magnitude of the diffusion tensor (L), linear tensor (Cl), planar tensor (Cp), spherical tensor (Cs), relative anisotropy (RA), axial diffusivity (AD) and radial diffusivity (RD)^10^; each one representing a single global measure of the whole-brain. Figure 1A shows the algorithm for measuring the DTI-derived tensor metrics.

### Statistical analysis

#### Study design

The study was considered a Phase II aimed to determine the capacity of DTI-derived biomarkers to distinguish between people with cancer and those without.^20^

#### Sample size

Considering our predictive model to discriminate between normal brains *vs*. brains infiltrated with GBM underwent a diagnostic performance assessment, the adequacy of the sample size to expect validity from our results was based on matching this phase with the summarized list of computed sample sizes needed for an exploratory retrospective study reported by Obuchowski *et al*.^21^, at least 10 diseased patients and 10 control patients were required to maintain statistical validation in a diagnostic test evaluation where the type I error rate was set at 0.05, type II error rate was ≤ 0.10, and power ≥ 0.90. Our study included 27 patients and 34 controls.

#### Multivariate DA

We ran a DA, which was optimal under the same conditions where Manova was optimal; then attempted to detect any deviation from Manova assumptions that might distort the tests of statistical significance.^22^ We assessed the normality of the distribution of the DTI-derived scores using the Kolmogorov–Smirnov’s and Shapiro-Wilk normality tests^23^; eliminated significant outliers, evaluated multivariate normality and linearity, and tested the homogeneity of variance-covariance matrices using the Box’s M test.^24^ Considering the similarity of the tensor-metric formulae, we ran scatterplots and correlations to check the strength of correlations among the dependent variables in order to detect the presence of multicollinearity and singularity (Table 1). Partial correlation analyses were carried out to calculate the Pearson’s correlation coefficient (r) controlling for the effect of age, gender and clinical diagnosis. The strength of the linear relationship corresponding to each correlation coefficient value was interpreted as very strong (at least of 0.8), moderately strong (0.6 up to 0.8), fair (0.3 up to 0.6) and poor (less than 0.3). A squared r value represented the coefficient of determination, the proportion of variance that each two compared variables had in common.^25^

We applied the stepwise method in *DA*, it considered the value of Wilk’s lambda and changing criteria: minimum partial F to enter of 3.84 and minimum partial F to remove of 2.71.^22^ Continuous variables were included with the predictive aim to identify specific tensor-metric attributes in GBM and normal brains. The dependent variable (DV) used in the DA was the clinical diagnosis, which classified subjects as patients or controls. The independent variables (IVs) included 11 DTI-derived tensor metrics: MD, FA, p, q, L, Cl, Cp, Cs, RA, RD and AD, and the patients’ age (in years). The effectsize measure for discriminant analysis was calculated using the squared canonical correlation as the equivalent of the R^2^ in regression.26 By convention, effect sizes of 0.02, 0.15, and 0.35 are termed small, medium, and large, respectively.^27^ For all analyses, statistical significance was indicated by a *p-value* < 0.05.

#### Diagnostic model evaluation

The cross-validated contingency Table generated by the DA was used to evaluate the diagnostic performance of the DA model. We reported values of sensitivity, specificity, positive and negative likelihood ratios, and positive and negative predictive values, with their corresponding confidence intervals (CI). Evaluation of the diagnostic tests followed the Standards for Reporting of Diagnostic Accuracy (STARD) initiative.^28^

#### Software

All analyses were carried out using the IBM^®^ SPSS^®^ Statistics software (version 22.0.0.0 IBM Corporation; Armonk, NY, USA). Diagnostic performance was assessed using MedCalc^®^ (version 12.3.0.0 MedCalc Software bvba, Mariakerke, Belgium).

## Results

### Subjects and MRI acquisition

The study was conducted in 61 subjects; 27 patients: 13 females (mean age 50.0 ± 15.400 years, range 31–73 years) and 14 males (mean age 46.93 ± 15.403 years, range 18–78 years); and 34 controls: 26 females (mean age 41.04 ± 22.37 years, range 21–80 years) and 8 males (mean age 42.88 ± 21.89 years, range 24–72 years). The eleven DTI tensor-maps plus the age (per subject) added up 732 measurements included in the analyses. Figure 1 B-E shows examples of some of the MR sequences and tensor-metric maps used in the data analyses.

### Partial correlation analyses

**FIGURE 2.**
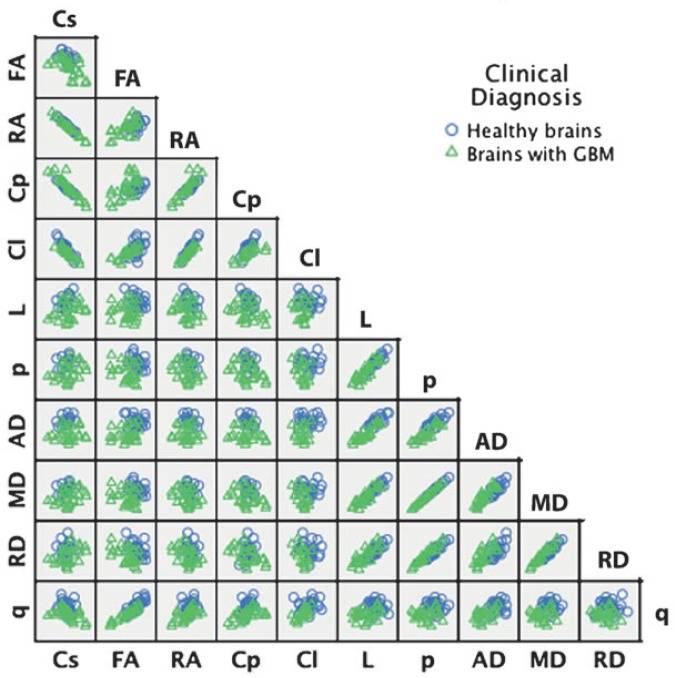
Scatter matrix of the data variables grouped by diagnosis.

A scatterplot showed no serious violation of the assumptions of linearity, homoscedasticity, and outliers. Among 55 pairs of bivariate correlations, we found only 15 with a significantly very strong (at least 0.8) r value: Cs⇔RA (−), Cs⇔Cp (−), Cs⇔L (−), FA⇔q (+), RA⇔Cp (+), RA⇔Cl (+), L⇔p (+), L⇔AD (+), L⇔MD (+), L⇔RD (+), p⇔AD (+), p⇔MD (+), p⇔RD (+), AD⇔MD (+), and MD⇔RD (+). Table 1 and Figure 2 depict correlation values and the scatterplot of the eleven tensor-metrics.

### Discriminant analysis

Although some r values were calculated at > 0.8, we included all variables in the DA, as we found evidence the stepwise variant of this method protects against multicollinearity and singularity;^29^ a brief explanation is presented in the discussion section. The assumption of homogeneity of variance-covariance matrices was interpreted as significant (Box’s M value = 35.110, F = 5.317, df (6, 9087.738), p = < .001). In the stepwise statistics, at each step, the best variable that minimized the overall Wilks’ Lambda was entered: AD was entered at Step 1, F = 42.052 (1, 36) p < .001; Cl entered at Step 2, F = 29.609 (2, 35) p < .001; and Cs entered at Step 3, F = 23.672 (3, 34) p < .001.

DA revealed one discriminant function that significantly differentiated the normal brains and GBM brains: Wilks’ λ = 0.324, χ^2^ (3) = 38.907, p < .001. By indicating the significance of the discriminant function, Wilks’ lambda provided a moderate proportion of total variability not explained by the model of 10.49%. A canonical correlation of 0.822 suggested the model explains 67.56% of the variation in the grouping variable.

### Summary of discriminant functions

The tests of equality of group means provided statistical evidence of significant differences between means of normal brains and brains with GBM in 9 of the IVs, with AD producing the highest F’s value; Table 2 depicts the means, standard deviations (SD) and F’s tests values (between-groups multivariate analysis).

*Standardized canonical discriminant function coefficients* showed an index of the importance of each predictor for diagnosis with the sign indicating the direction of the relationship. A significant increase in values of Cs (spherical tensor), Cl (linear tensor) and AD (axial diffusivity) were the strongest diagnostic predictors. The variable coefficients stood out (for these data) as those that strongly predicted allocation to the normal-brain or tumor-brain group. The coefficient score decrement was proportional to less successful diagnostic predictors (Table 3A).

*Structure Matrix Data* provided another way of indicating the relative importance of the diagnostic predictors by showing the correlations (Pearson coefficients) of each variable with each discriminate function. Many researchers consider the structure matrix correlations more accurate than the standardized canonical discriminant function coefficients.^26^ By identifying the largest loadings for each discriminate function, different patterns of loading variables can be seen. We found AD, MD, p, q, Cl, RD and FA, as the functions that best discriminate between normal brains and brains with tumor. A value of 0.30 was considered as the cutoff between important and less important variables (Table 3B).^30^

*The canonical discriminant function coefficients Table* showed the unstandardized coefficients (b) that were used to create the discriminant function (equation), they operated just like a regression equation, allowing us to build a predictive model of brain status:

Brain status (normal brain vs. tumor infiltration) = −48.295 +11,443.557 (axial diffusivity, AD) +105.124 (longitudinal tensor, Cl) +26.804 (spherical tensor, Cs)

The discriminant function coefficients (b) indicated the partial contribution of each variable to the discriminate function controlling all other variables in the equation (Table 3C).

*The group centroids values* described each group in terms of its profile, using the group means of the predictor variables called centroids. The cutoff value was defined as the mean of the two centroids; if the discriminant score of the function of a new case was less than or equal to the cut-off, the case was classed as 1 (brain with tumor), whereas if it was above the cut-off, it was classed as 0 (normal brain). In our study, normal brains had a mean of 1.483 while brains with GBM produced a mean of −1.334; the cut-off for the function at group centroids showed a calculated value of 0.149.

For the final part of the DA we performed a classification phase using the cross-validated set of data to present the power of the discriminant function. These results revealed that 92.7% of patients were classified correctly into “normal brain” or “brain with GBM” groups, this value corresponded to the overall predictive accuracy of the discriminant function. Additional results of diagnostic tests performance including the 95% confidence intervals (C.I.) showed: sensitivity = 100.00 (80.49 – 100.00); specificity = 87.50 (67.64 – 97.34); (+) likelihood ratio = 8.00 (82.78 – 23.06); (−) likelihood ratio = 0.00 (−); (+) predictive value = 85.00 (62.11 – 96.79); and (−) predictive value = 100.00 (83.89 – 100.00).

**FIGURE 3.**
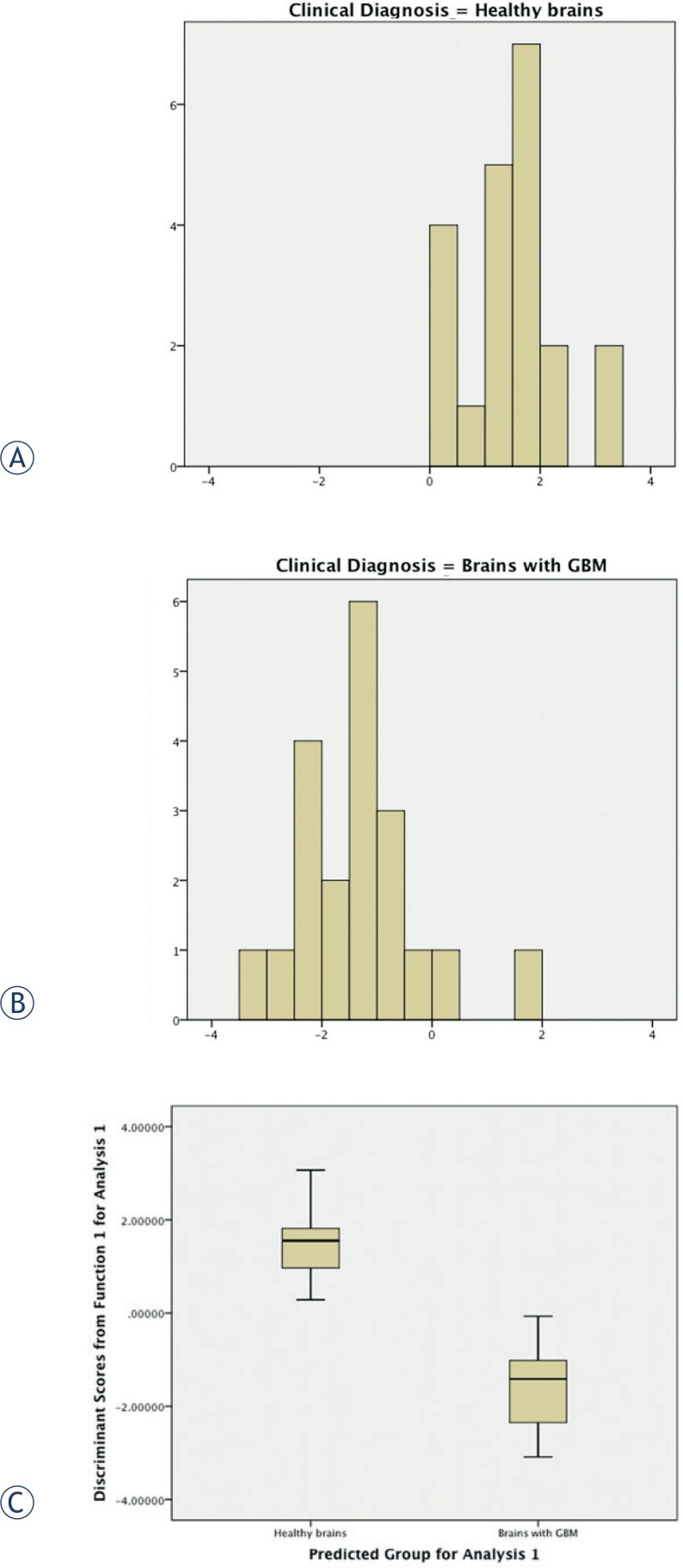
Visual demonstration of the effectiveness of the discriminant function. **(A),** histograms showing the distribution of discriminant scores for normal- and tumor-brains. **(B),** box plots of the average D scores. Both kinds of plots illustrate the distribution of the discriminant function scores for each group. The box-plots depict a visual demonstration of the excellent discrimination of the model by showing no overlap between groups.

The average discriminant (D) scores for each group and the group centroids were used as visual demonstrations of the effectiveness of the discriminant function. Histograms and box plots of the average D scores for each group were used as graphical demonstrations of the effectiveness of the discriminant function, the absence of overlap of the plots revealed an excellent discrimination (Figure 3 A–B).

## Discussion

The lack of consensus regarding which DTI-derived tensor metrics are the most meaningful^31^, and the scarce information about their diagnostic abilities, compelled us to evaluate whether a *global* approach might have clinical applicability. We consider our study an introduction to the method and proof-of-principle that a *global* approach using selected DTI-derived tensor metrics can differentiate normal brains from brains infiltrated with GBM, the selected metrics may function as biomarkers assembling a predictive model of tumor infiltration.

The relevant findings in our study showed that a multivariate DA of global measurements excluded the pair-wise comparisons from conventional tumor-region evaluations; the assembled statistically significant discriminant model of tumor brain impairment (for these data) needed only three *global* DTI-derived metrics: AD, Cl, and Cs.

Some advantages of a global approach using DTI metrics need to be mentioned: it decreases the bias associated with manual placement of a region of interest encompassing tumor regions; the tumor and edema regions are implicitly included in the evaluation; lesions not perceived by the radiologist’s eye on conventional sequences would be included in a global assessment; it may avoid problems associated with partial volume effects, and inaccurate image coregistrations; DTI biomarkers can be applied to other tumors/neurological diseases; its acquisition does not need contrast, and its post processing method can be semiautomatic; these facts broad the clinical applicability with no significant increase in the cost of MRI examinations.

The selected biomarkers in our final model deserve a brief explanation: AD depicted the main influence (larger value of its b unstandardized coefficient); it represents the directional diffusivity describing the microscopic water movement parallel to axonal tracts. AD is one of the best biomarkers in the diagnosis of enhancing rim in GBM, but not for other tumor regions^10^; (this fact provides evidence that a *regional* measurements may not be the most effective way to use DTI metrics in brain tumor imaging).^14^ AD has been studied in animal models of encephalomyelitis of the spinal cord^32^,^33^, in unfixed *ex vivo* human brains with multiple sclerosis34, in a model of axonal injury caused by stroke^35^, and in optic neuritis. Cl and Cs on the other hand, along with FA, have been reported among the biomarkers with best overall performance in differentiating the cystic cavity in abscess from GBM.^13^ They show best diagnostic performance in the detection of normal-appearance white matter (NAWM) and the cystic cavity in brains with GBM.^10^ Cs represents spherical normalized coordinates of a nonorthogonal DTI-derived tensor for each voxel, and Cl corresponds to the linear case.^36^

Several limitations in this study need to be addressed: because there have not been studies investigating a whole set of tensor metrics (not only FA and ADC) in a global approach^1^,^37^,^38^, it is difficult to compare our results with others in the literature. Further studies might include comparisons with other brain tumors, the influence of variables like radiation necrosis, inflammatory and demyelinating diseases; and tumor infiltration categories such as post-surgery and post-radiotherapy; all of them were beyond the scope of this study. A concern of using DTI-metric values with high correlations (correlations up 0.8 or 0.9), as we observed in our data, might be raised because in those situations one variable is a near-linear combination of the other variable (the variable provides inform ation that is redundant to the information available in one or more of the others, making matrix inversion unreliable).^29^ The usual solution is a deletion of the redundant variable, however, because we have a compelling theoretical reason to retain all variables in this study (to evaluate the simultaneous discriminant ability of 11 global tensor metrics), the IBM^®^ SPSS^®^ Statistics software protects against multicol-linearity and singularity through computation of pooled within-cell tolerance (1-squared multiple correlation, SMC) for each variable. SMC is the squared multiple correlation of a variable where it serves as the dependent variable (DV) with the rest as independent variables (IV) in multiple correlation. Variables with insufficient tolerance are deleted from the analysis; this procedure is a part of the stepwise method in DA.^22^ Our discriminant model was able to explain a significant proportion of the variability in the data (67.56%), but may still have some errors in predicting individual diagnosis, so model validation should be done in subsequent studies. We acknowledge the linking of tensor-metric values with the axonal-integrity status represents an oversimplification with respect to what is happening in brains with GBM, where complex tissue changes occur and affect water diffusivity: density of fiber, average diameters, degree of myelination, directional similarity, cellularity, viscosity, permeability, and histologic architecture; the DTI-tensor values are the effects of the summation of all these microstructural barriers.^13^

Several questions remain unanswered, for example, what is the relation of these tumor-DTI biomarkers with those of MR perfusion and spectroscopy? What is the association of DTI-biomarkers with the pattern of relapse and extension of resection in GBM? So far, only one study, to the best of our knowledge, has correlated a few *regional* DTI-tensor metrics with the survival of patients with GBM^39^; thus the clinical value of global DTI-metrics in predicting the overall survival has yet to be determined. As a phase II study, our research line will look for a sequel, applying the proven concepts in the follow-up of tumor-infiltration categories (post-surgery, post-radiotherapy, etc.) and in differential diagnoses (primary brain tumors vs. metastasis vs. demyelinating diseases).

## Conclusions

Although we cannot affirm the superiority of global vs. regional DTI-derived tensor metrics in the evaluation of GBM yet, we can ascertain with certainty that there is an immediate clinical applicability of these biomarkers in assembling statistically significant predictive models able to announce the conversion of normal tissue to tumor infiltrated tissue before the conventional MR sequences show conspicuous findings. These principles could easily be extended to other neurological diseases. A first step in the advanced evaluation of brain tumors might include a global measurement of DTI-biomarkers able to pick up major infiltration zones. Due to the large number of variables (qualitative and quantitative) that must be analyzed in contemporary brain MRI by radiologists and neuroscientists conducting research on novel imaging biomarkers; multivariate techniques, like DA, may help in the generalization of knowledge beyond one setting.

## Figures and Tables

**TABLE 1.** Correlations of tensor metrics, controlled for the effect of diagnosis, age and gender

**Tensor metric**												
		**Cs**										
**FA**	Pearson’s R	−.552	**FA**									
	*p*-value	< .001										
**RA**	Pearson’s R	−1.000	.557	**RA**								
	*p*-value	< .001	< .001									
**Cp**	Pearson’s R	−.937	.584	.912	**Cp**							
	*p*-value	< .001	< .001	< .001								
**Cl**	Pearson’s R	−.898	.541	.943	.673	**Cl**						
	*p*-value	< .001	< .001	< .001	< .001							
**L**	Pearson’s R	.211	.075	−.175	−.191	< .001	**L**					
	*p*-value	.165	.596	.256	.213	.999							
**p**	Pearson’s R	.195	−.079	−.183	−.194	.006	.890	**p**				
	*p*-value	.205	.580	.240	.214	.972	< .001					
**AD**	Pearson’s R	.034	.209	−.008	−.002	.106	.882	.880	**AD**			
	*p*-value	.826	.137	.958	.989	.508	< .001	< .001				
**MD**	Pearson’s R	.195	−.078	−.183	−.193	.007	.892	1.000	.881	**MD**		
	*p*-value	.206	.589	.241	.214	.968	< .001	< .001	< .001			
**RD**	Pearson’s R	.213	−.226	−.209	−.149	−.105	.815	.973	.779	.973	**RD**	
	*p*-value	.151	.103	.163	.316	.502	< .001	< .001	< .001	< .001		
**q**	Pearson’s R	−.306	.804	.310	.339	.328	.403	.281	.627	.284	.214	**q**
	*p*-value	.031	< .001	.030	.016	.026	.003	.046	< .001	.044	.116	

AD = axial diffusivity; CI = linear tensor; Cp = planar tensor; Cs = spherical tensor; FA = fractional anisotropy; L = the total magnitude of the diffusion tensor; MD = mean diffusivity; p = pure isotropic diffusion; q = pure anisotropic diffusion; RA = relative anisotropy; RD = radial diffusivity

**TABLE 2.** Multivariate analysis (between-groups) of diffusion tensor imaging (DTI)-derived tensor metrics and age showing the statistical differences between means of normal-brain and brain-with- as glioblastoma multiforme (GBM) groups for the independent variables included in the analysis

**Variable**	**Healthy brains**		**Brains with GBM**		**Wilks’ Lambda**	**F test**	**p-value**
	**Mean**	**SD**	**Mean**	**SD**			
Cs (spherical tensor)	.747091	.026938	.768395	.042299	.915	3.341	.076
FA (fractional anisotropy)	.287029	.011517	.254082	.026761	.607	23.341	< .001
RA (relative anisotropy)	.233436	.025491	.209370	.033559	.855	6.088	.018
Cp (planar tensor)	.138366	.013823	.136635	.036218	.999	.036	.850
Cl (linear tensor)	.114543	.013991	.098463	.011930	.711	14.621	.001
L (total magnitude of the diffusion tensor)	.002277	.000087	.002117	.000147	.691	16.077	< .001
p (pure isotropic diffusion)	.002107	.000077	.001959	.000134	.681	16.893	< .001
AD (axial diffusivity)	.001548	.000044	.001399	.000087	.461	42.052	< .001
MD (mean diffusivity)	.001217	.000044	.001132	.000078	.685	16.526	< .001
RD (radial diffusivity)	.001051	.000050	.000997	.000078	.852	6.237	.017
q (pure anisotropic diffusion)	.000452	.000036	.000367	.000047	.483	38.529	< .001
Age	40.333	21.502	47.150	15.187	.965	1.294	.263

SD = standard deviation

**TABLE 3.** Independent variables included in the discriminant analysis. A, ordered by their Standardized Canonical Discriminant Function Coefficients (variables with larger coefficients stand out as those that strongly predict allocation to each diagnosis). B, Within-groups correlation matrix depicts the participant variables ordered by absolute size of correlation (Pearson coefficients) within function. The largest loadings for each discriminate function (AD was the largest) suggest the preference of diffusivity values that discriminates between normal- and brain-tumor groups. A value of 0.30 is considered as the cut-off between important and less important variables, notice that variables with (*) were not used in the analysis. C, unstandardized coefficients used to create a discriminant function operating just like a regression equation. Coefficients indicate the partial contribution of each variable to the discriminate function controlling for all other variables in the equation

**A**		**B**		**C**	

**Standardized Canonical Discriminant Function Coefficients**		**Structure Matrix**		**Canonical Discriminant Function Coefficients**	

**Variable**	**Function**	**Variable**	**Function**	**Variable**	**Function**
	**1**		**1**		**1**
Cl (linear tensor)	1.361	AD (axial diffusivity)	.748	AD (axial diffusivity)	11443.557
Cs (spherical tensor)	.962	MD (mean diffusivity)*	.568	Cl (linear tensor)	105.124
AD (axial diffusivity)	.806	p (pure isotropic diffusion)*	.566	Cs (spherical tensor)	26.804
		L (total magnitude of the diffusion tensor)*	.553	(Constant)	− 48.295
		q (pure anisotropic diffusion)*	.533		
		Cl (linear tensor)	.441		
		RD (radial diffusivity)*	.427		
		FA (fractional anisotropy)	.320		
		RA (relative anisotropy)*	.278		
		Cs (spherical tensor)	− .211		
		Age*	.124		
		Cp (planar tensor)*	.020		
